# DFT investigation of boron- and zinc-doped C24 fullerenes as efficient nanosensors for molly detection

**DOI:** 10.1038/s41598-025-32724-1

**Published:** 2025-12-19

**Authors:** Mohammed Ghazwani, Umme Hani

**Affiliations:** Department of Pharmaceutics, College of Pharmacy, King Khalid University, 62223 Al Faraa, Abha, Saudi Arabia

**Keywords:** Molly, Doped fullerenes C_24_, Colorimetric sensing, DFT, Electrochemical sensor, MDMA detection, Chemistry, Materials science, Nanoscience and technology

## Abstract

Reliable detection of 3,4-methylenedioxymethamphetamine (Molly) remains a major analytical challenge due to its widespread recreational use and high risk of combination with toxic substances, as well as the limitations of conventional laboratory methods such as GC-MS and Raman spectroscopy. This study employs Density Functional Theory (DFT), Time-Dependent DFT (TD-DFT), Quantum Theory of Atoms in Molecules (QTAIM), Natural Bond Orbital (NBO), and Non-Covalent Interaction (NCI) analyses to design and evaluate pristine and doped C24 fullerenes (BC23 and ZnC23) as potential colorimetric and electrochemical nanosensors for Molly detection. Computational findings reveal that boron and zinc doping enhance structural stability, with cohesive energies increasing from 149 kcal mol^− 1^ (C_24_) to 194 kcal mol^− 1^ (BC_23_) and 188 kcal mol^− 1^ (ZnC_23_). Electronic analysis shows that doping reduces the HOMO-LUMO gap from 6.12 eV (C_24_) to 5.68 eV (BC_23_) and 5.24 eV (ZnC_23_), improving reactivity and charge transfer. The BC_23_@Molly complex (Conformer 4) exhibited the highest adsorption energy (− 18.19 kcal mol^− 1^) and a remarkable redshift in λmax from 444 to 660 nm, confirming its superior colorimetric sensitivity. Conversely, the ZnC_23_@Molly complex (Conformer 6) demonstrated the fastest recovery time (3.8 × 10^− 4^ s) and highest electrical conductivity (2.78 × 10^9^ A m^− 2^), identifying it as the most efficient electrochemical sensor. QTAIM and NCI analyses confirmed the presence of medium-strength hydrogen bonding and dispersive interactions, while NBO data revealed strong π→π (21.13 kcal mol^− 1^)* and LP→π (55.97 kcal mol^− 1^)* transitions in BC_23_@Molly. Collectively, these results establish BC_23_ as the most effective colorimetric sensor and ZnC_23_ as the optimal electrochemical sensor for rapid, sensitive, and field-deployable Molly detection.

## Introduction

Molly (3,4-methylenedioxymethamphetamine) is a synthetic psychoactive drug known for its stimulant and hallucinogenic effects. It is often associated with feelings of euphoria, emotional warmth, and sensory enhancement, making it popular in party and festival settings. However, identifying Molly is crucial because it is frequently adulterated with dangerous substances like methamphetamine, bath salts, or synthetic cathinones, posing serious health risks, including overdose, organ failure, and even death^1–3^. Common methods to identify Molly include gas chromatography-mass spectrometry (GC-MS), high-performance liquid chromatography (HPLC), Raman spectroscopy, and colorimetric reagent tests. GC-MS and HPLC are highly accurate but require expensive equipment, trained personnel, and laboratory settings, making them impractical for field use^4,5^. Raman spectroscopy offers portable versions but still involves costly devices and skilled operation^6^. Colorimetric tests (e.g., Marquis reagent) are more accessible and inexpensive but lack specificity and can give false positives or negatives, especially when cutting agents are present^7^.

Although traditional analytical techniques (GC-MS, HPLC, and Raman spectroscopy) remain the gold standard for drug detection due to their accuracy, their limitations create an urgent need for simpler, more accessible, and more accurate methods for molecular identification, especially in non-laboratory settings. In this regard, computational methods offer a rapid, cost-effective, and predictive alternative for screening and designing sensor materials prior to laboratory synthesis. These methods provide molecular-level insights into the adsorption behavior, charge transfer, and optical responses, helping to identify the most promising sensor candidates, thereby reducing the number of experimental iterations required.

The use of colorimetric and electrochemical sensors has received much attention in recent research due to their sensitivity, rapid response, and potential for in-situ application^8,9^. These sensors are often constructed using nanomaterials, particularly carbon-based nanomaterials, because of their unique electrical, optical, and chemical properties^10^. Among these, graphene, carbon nanotube, and fullerenes have shown great promise as active components in sensor design. Notably, fullerenes (especially the C24 fullerene) have gained increasing interest. Compared to other carbon nanostructures, C_24_ fullerene offers several advantages, including a highly symmetrical and compact cage structure, exceptional electron-accepting capacity, and a high surface-to-volume ratio, which enhances molecular interactions. These properties contribute to improved sensitivity, selectivity, and stability of sensors using C_24_, making it a desirable candidate for developing advanced colorimetric and electrochemical drug detection platforms^11,12^.

However, it is important to note that pristine fullerenes also present challenges, such as limited solubility in aqueous media and potential cytotoxic effects that may restrict their direct biomedical applications. Several reports have documented reduced selectivity and non-specific adsorption behavior in fullerene-based sensors, highlighting the need for controlled doping or functionalization to overcome these issues^13^.

In this regard, N.A. Tukadiya et al. introduced fullerene C_24_ and its derivatives as effective candidates for glucose sensing^14^. Similarly, S. Tayebi-Moghaddam et al. highlighted the potential of C_24_ fullerene as a sensor for detecting acrylamide, and M.T. Baei et al. demonstrated the sensitivity of C_24_-based structures in detecting nicotine molecules^15,16^. Moreover, several studies have shown that doping fullerenes with elements such as boron and zinc significantly enhances their electronic properties, such as charge distribution and adsorption capability^17,18^. This enhancement is an important advantage, as it can improve the sensitivity and selectivity of these structures when used as sensors. Given the importance of accurately detecting the drug Molly and considering the demonstrated sensing capabilities of fullerene C_24_, this study investigates the interaction between Molly and both pristine and doped forms of C_24_ fullerene, specifically boron- and zinc-doped structures.

The goal is to design fast, efficient, and sensitive sensors for molar detection using computational methods. Today, computational screening has emerged as an essential tool in the synthesis of drug diagnostic sensors and the design of nanomaterials. The use of advanced theoretical frameworks such as density functional theory (DFT), time-dependent DFT (TD-DFT), and quantum theory of atoms in molecules (QTAIM) allows for systematic investigation of molecular stability, reactivity, and adsorption behavior prior to experimental validation^19–21^.

Furthermore, Density Functional Theory (DFT) investigations have identified several intrinsic limitations of fullerene-based systems, such as self-interaction errors, charge delocalization inaccuracies, and difficulties in predicting weak van der Waals interactions accurately^22^. These challenges are partly mitigated through dopant incorporation, as demonstrated by recent DFT analyses of boron-, nitrogen-, and transition-metal-doped nanocages, which exhibit improved charge transfer and adsorption behavior^23^.

This predictive approach significantly reduces the cost and time associated with trial-and-error synthesis by identifying the most promising molecular configurations and dopant effects in silico. In the field of drug detection sensor synthesis, computational screening helps in the rational design of diagnostic sensors, catalysts, and nanocarriers by elucidating the relationships between electronic structure and biological activity. In the present work, computational screening was used not only to design and optimize C_24_ fullerene-based sensors for Molly detection but also to provide insights into how these nanostructures could potentially function as active components in drug delivery or therapeutic systems. This approach highlights the pivotal role of computational modeling in guiding future experimental synthesis and application of fullerene-derived materials in pharmaceutical sciences.

## Computational details

**Fig. 1:**
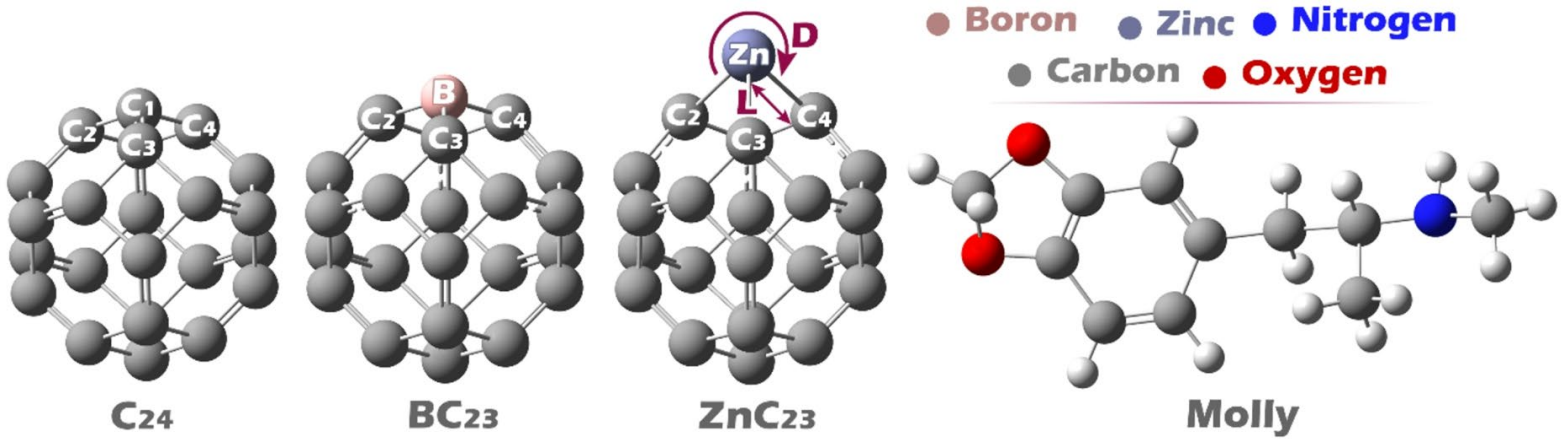
Optimized geometry of each of the studied structures.

First, all structures, including C24 fullerene, its B/Zn-doped derivatives, and Molly, were modeled using GaussView 6.0 and then geometrically optimized using Gaussian 09 W software^24^. Dopant atoms (B and Zn) were modeled as isolated atomic species within the Gaussian framework to simulate substitutional doping at a single carbon site in the C_24_ nanocage. Geometric optimizations were performed in the water phase (simulated via the CPCM solvation model) using Gaussian 09 W (Revision B.01) with the ωB97XD functional and the 6-31G(d) basis set (Fig. 1)^25,26^.

The CPCM model was chosen because it provides a reliable balance between computational efficiency and accuracy for aqueous-phase systems, particularly for neutral organic analytes and fullerene-based nanostructures. While SMD solvation could offer more comprehensive treatment of solute-solvent interactions, CPCM has been widely validated for adsorption and charge-transfer studies involving nonpolar to moderately polar systems, making it suitable for the current work. Future studies may extend this model to SMD or explicit hybrid QM/MM frameworks to further assess solvation and stability in biological media.

All optical property analyses were performed using the Time-Dependent DFT (TD-DFT) method at the same ωB97XD/6-31G level of theory^27^. This combination was selected based on its proven ability to reproduce excitation energies and optical spectra in conjugated carbon systems with minimal computational cost. The ωB97XD functional includes long-range and dispersion corrections, ensuring accurate treatment of weak non-covalent interactions critical for molecular adsorption and colorimetric response^28,29^.

UV-Vis spectra were generated and analyzed using the open-source GaussSum software (version 3.0; available at https://gaussum.sourceforge.net)^30^.

Additionally, quantum theory of atoms in molecules (QTAIM) was employed to analyze bond critical points (BCPs), providing insights into the nature and strength of interactions between Molly and the C_24_-based nanostructures^31^.

The cohesive energy (E_Coh_) was calculated using Eq. (1).

1 $${E}_{Coh}=-\left({{E}}_{{t}{o}{t}}-\sum\limits_{{i}}{{n}}_{{i}}{{E}}_{{i}}\right)/{n}$$

In this equation: Etot = Total energy of the optimized structure (e.g., C_24_ fullerene); Ei = Energy of an isolated atom of type i (e.g., C, B, Zn); ni = Number of atoms of type i in the system; n = Total number of atoms in the system (e.g., *n* = 24 for C_24_)^32^.

The energy gap (HLG), chemical softness (S), chemical hardness ($\:{\upeta\:}$), and chemical potential (µ) were calculated using Eqs. (2–5)^33–35^.

2 $$\:\mathrm{H}\mathrm{L}\mathrm{G}=\left|{\mathrm{E}}_{\mathrm{H}\mathrm{O}\mathrm{M}\mathrm{O}}-{\mathrm{E}}_{\mathrm{L}\mathrm{U}\mathrm{M}\mathrm{O}}\right|$$

3 $${{\eta }} = {{\left( { - {\mathrm{E}}_{{{\mathrm{HOMO}}}} - \left( { - {\mathrm{E}}_{{{\mathrm{LUMO}}}} ~} \right)} \right)} \mathord{\left/ {\vphantom {{\left( { - {\mathrm{E}}_{{{\mathrm{HOMO}}}} - \left( { - {\mathrm{E}}_{{{\mathrm{LUMO}}}} ~} \right)} \right)} 2}} \right. \kern-\nulldelimiterspace} 2}$$

4 $$\:{\upmu\:}=-(-{\mathrm{E}}_{\mathrm{H}\mathrm{O}\mathrm{M}\mathrm{O}}+(-{\mathrm{E}}_{\mathrm{L}\mathrm{U}\mathrm{M}\mathrm{O}}\left)\right)/2$$

5 $$\:{S}=1/2{\upeta\:}$$

E_HOMO_ = Energy of highest occupied molecular orbital (HOMO), E_LUMO_ = Energy of lowest unoccupied molecular orbital (LUMO). The maximum charge transferred (ΔNmax) and charge transfer based on electrophilicity (ECT) were calculated using Eqs. (6) and (7), respectively.

6 $$\:{\varDelta\:{N}}_{{m}{a}{x}}=-\mu / \eta$$

7 $$\:{E}{C}{T}={\left({{\Delta\:}{N}}_{{m}{a}{x}}\right)}_{{\alpha\:}}-{\left({{\Delta\:}{N}}_{{m}{a}{x}}\right)}_{{\beta\:}}$$

$\:{\left({{\Delta\:}N}_{max}\right)}_{\alpha\:}$ represents the maximum charge transferred by the complex and $\:{\left({{\Delta\:}N}_{max}\right)}_{\beta\:}$ represents the maximum charge transferred by the sensor. A positive ECT value indicates no net electron transfer from the sensor to Molly (the sensor acts as an electron donor) while a negative ECT value indicates electron transfer from Molly to the sensor (the sensor acts as an electron acceptor)^36,37^.

Recovery time (τ) and electrical conductivity ($\:{\upsigma\:}$) were calculated using Eqs. 8 and 9, respectively.

8 $$\:{\tau\:}={{V}}_{0}^{-1}\times\:\mathrm{e}\mathrm{x}\mathrm{p}\left(-\frac{{{E}}_{{a}{d}{s}}}{{{k}}_{{B}}{T}}\right)$$

9 $$\:{\upsigma\:}={A}{{T}}^{3/2}{{e}}^{(-{H}{L}{G}/2{K}{T})}$$

In these equations: A = Richardson constant (6 × 10^5^ A m^− 2^), k_B_= Boltzmann constant, E_ads_= adsorption energy, V_0_ = attempt frequency (10^14^ s^− 1^), and, T = temperature (298 K)^38,39^.

The adsorption energy (E_ads_) for each complex was calculated using Eq. 10.

10 $$\:{{E}}_{{a}{d}{s}}={{E}}_{\left({R}-\right){C}24@{M}{o}{l}{l}{y}}-\left({{E}}_{{M}{o}{l}{l}{y}}+{{E}}_{\left({R}-\right){C}24}\right)+{{E}}_{{B}{S}{S}{E}}$$

In these equations: E_(R−)C24@Molly_= Total energy of the optimized complex (Molly adsorbed on R-functionalized C_24_, where R = B, Zn, or pure C_24_), E_Molly_= Energy of the isolated Molly molecule (optimized geometry), E_(R−)C24_= Energy of the isolated functionalized C_24_ fullerene (R = B/Zn or pure C_24_) in its optimized form, and E_BSSE_= Basis Set Superposition Error correction^40,41^.

These calculations aimed to systematically investigate the adsorption behavior, electronic properties, and sensing potential of C_24_ fullerene and its functionalized derivatives toward Molly detection.

## Results and discussion

### Structural properties

#### Bond length and bond angles

In sensor design, considering bond length and bond angle is crucial because these structural parameters directly influence the electronic and chemical properties of the sensor material. One of the key aspects influenced by bond length and bond angle is the mobility of π electrons. Optimal bond lengths and bond angles can increase the overlap between adjacent p-orbitals, allowing the π-electrons to move more freely. This increased delocalization improves the electronic conductivity and sensitivity of the sensor^42,43^. Therefore, the bond length and bond angle for each of the designed structures were computationally studied, and the results are reported in Table 1.

**Table 1:** Bond length/angle in pristine C24 and its doping forms with B and Zn.

Structure	Bond lengths (Å)		Bond angles (°)	
C_24_	C1–C2	1.48	C2-C1-C4	89.99
	C1–C4	1.48	C2-C1-C3	119.97
	C1–C3	1.36	C3-C1-C4	119.93
BC_23_	B–C2	1.56	C2-N-C4	89.78
	B–C4	1.56	C2-N-C3	120.17
	B–C3	1.54	C3-N-C4	120.16
ZnC_23_	Zn–C2	2.05	C2-Zn-C4	67.27
	Zn–C4	2.05	C2-Zn-C3	93.27
	Zn–C3	2.02	C3-Zn-C4	93.27

Based on the data reported in Table 1, we can analyze the effect of bond lengths and bond angles on the structures of C_24_, BC_23_, and ZnC_23_ concerning their potential electronic behavior, particularly the mobility of π electrons.

In the pristine C_24_ structure, the bond lengths between carbon atoms (C1–C2 and C1–C4) are 1.48 Å, while the shorter C1–C3 bond is 1.36 Å. These values are consistent with typical carbon-carbon single and double bonds, suggesting partial delocalization of π electrons across the fullerene cage. The bond angles (C2–C1–C4: 89.99°, C2–C1–C3: 119.97°, C3–C1–C4: 119.93°) show a relatively symmetric geometry favorable for π orbital overlap, supporting effective electron delocalization and potentially good electrical conductivity.

In the BC13 structure, where a boron atom is substituted into the C_24_ cage, the bond lengths (B–C2 and B–C4: 1.56 Å; B–C3: 1.54 Å) are slightly longer than the C-C bonds in C_24_. This increase in bond length may reduce the overlap between adjacent p orbitals, slightly affecting the mobility of π electrons. However, the bond angles (89.78°, 120.17°, and 120.16°) remain close to those in C_24_, maintaining a geometry that still supports electron delocalization. The incorporation of boron, which is less electronegative than carbon, could also influence charge distribution in the system, possibly enhancing sensitivity to specific analytes without significantly disrupting π electron movement.

In the ZnC_23_ structure, where a zinc atom is doped into the fullerene framework, the bond lengths (Zn–C2 and Zn–C4: 2.05 Å; Zn–C3: 2.02 Å) are considerably longer than the C–C or B–C bonds in the other structures. This significant elongation indicates weaker orbital overlap, which may reduce π electron mobility in the vicinity of the zinc site. Additionally, the bond angles (C2–Zn–C4: 67.27°, C2–Zn–C3 and C3–Zn–C4: 93.27°) deviate substantially from the angles seen in C_24_ and BC_23_, reflecting a more distorted local geometry. Such angular distortion may further impact electronic delocalization, although zinc’s presence could introduce new electronic states that might enhance specific sensor functions depending on the target analyte.

Overall, comparing these structures reveals that doping with boron or zinc alters both bond lengths and bond angles relative to C_24_, leading to variations in molecular geometry that can influence π-electron behavior. While these changes may affect conductivity and sensitivity, they can also offer unique advantages in molecular recognition and selectivity depending on the application.

#### Cohesive energy

Cohesive energy is the amount of energy required to disassemble a solid into its atoms, reflecting the strength of the bonds holding the structure together^44^. It indicates the structural stability of a material; the higher the cohesive energy (in absolute value), the more stable the structure. In sensor design, considering cohesive energy is important because it ensures that the sensor material maintains its integrity under operational conditions. A stable structure is less likely to degrade, deform, or lose functionality, which is essential for reliable and long-lasting sensor performance^45,46^.

**Fig. 2:**
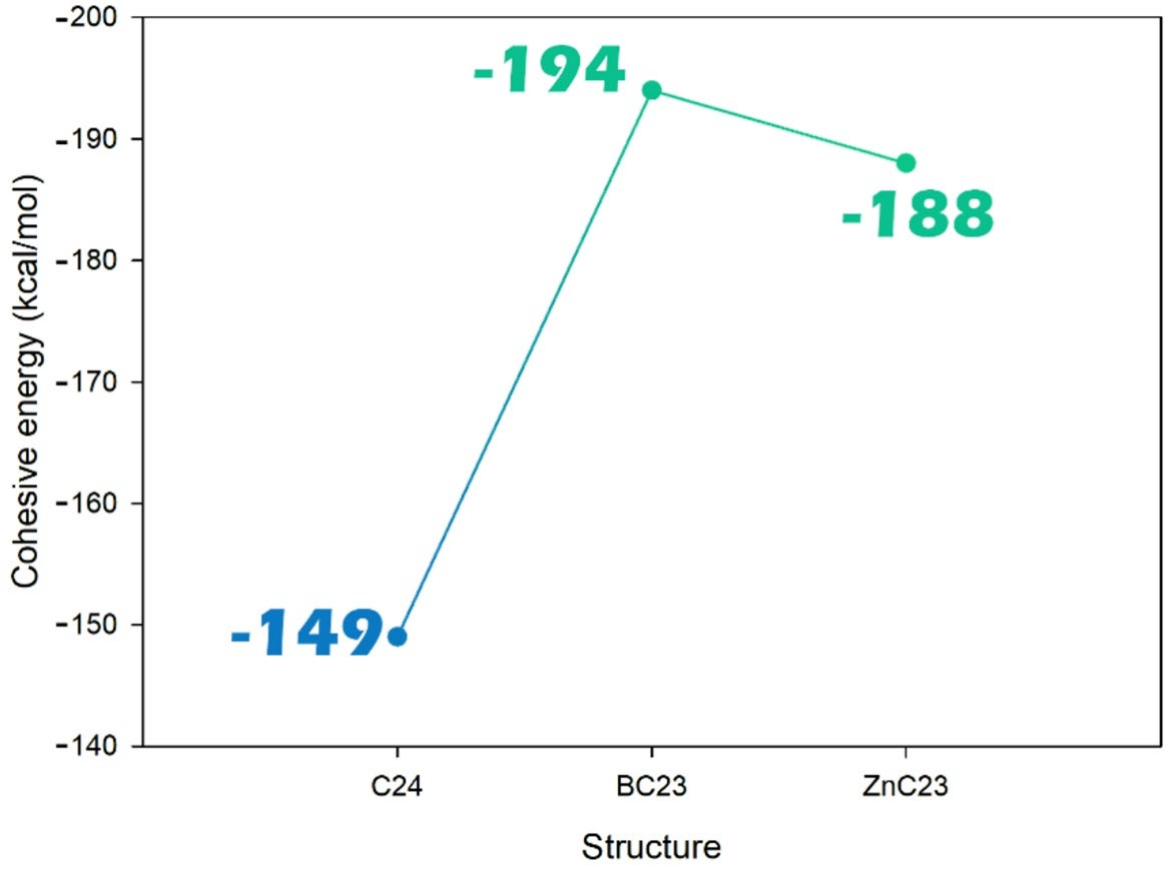
Cohesive energy change trend in pristine C24 after Zn and B doping.

The data in the Fig. 2 represent the cohesive energies of the C_24_, BC_23_, and ZnC_23_ structures. The C_24_ structure has a cohesive energy of − 149 kcal/mol, while the boron-doped BC_23_ and the zinc-doped ZnC_23_ structures have higher cohesive energies of − 194 kcal/mol and − 188 kcal/mol, respectively. The increase in cohesive energy upon doping suggests that both BC23 and ZnC23 are more structurally stable than the pristine C_24_. Among them, BC_23_ shows the highest cohesive energy, indicating the strongest atomic binding and overall structural integrity. This suggests that B and Zn doping not only modify the electronic properties of the fullerene but also enhance its structural stability, which is beneficial for designing robust and reliable sensor materials.

The observed higher cohesive energy of the BC_23_ structure compared to ZnC_23_ can be attributed to the nature of bonding introduced by the dopants. Boron, due to its smaller atomic radius and ability to form strong covalent B-C bonds with efficient p-p orbital overlap, enhances lattice cohesion and electron delocalization within the fullerene cage. In contrast, zinc forms relatively weaker Zn–C interactions of partial ionic character because of its filled d orbitals and larger size, which contribute less to overall structural binding. Therefore, despite zinc’s higher electronegativity, boron doping yields a more strongly bound and energetically stable framework, consistent with the observed increase in cohesive energy.

### MEP contour

Molecular Electrostatic Potential (MEP) contours play an important role in identifying the most reactive or interactive regions of a molecule. These contours map the distribution of electrostatic potential across the molecular surface, helping to predict where a molecule is most likely to interact with other species, such as analytes or target molecules in sensor applications. Regions with high negative potential are typically favorable for interactions with electrophilic (electron-deficient) species, while areas with positive potential are more likely to attract nucleophilic (electron-rich) species^47,48^. In MEP maps, the colors represent the strength and type of electrostatic potential. Red regions indicate areas of high negative potential, typically associated with electron-rich sites, such as lone pairs or π electrons. Blue regions show areas of high positive potential, often near hydrogen atoms or electron-deficient centers. Green, yellow, and orange areas represent intermediate potentials. Analyzing these color distributions allows for the investigation of the most likely interaction sites on molecules in the design of complexes^49,50^.

**Fig. 3:**
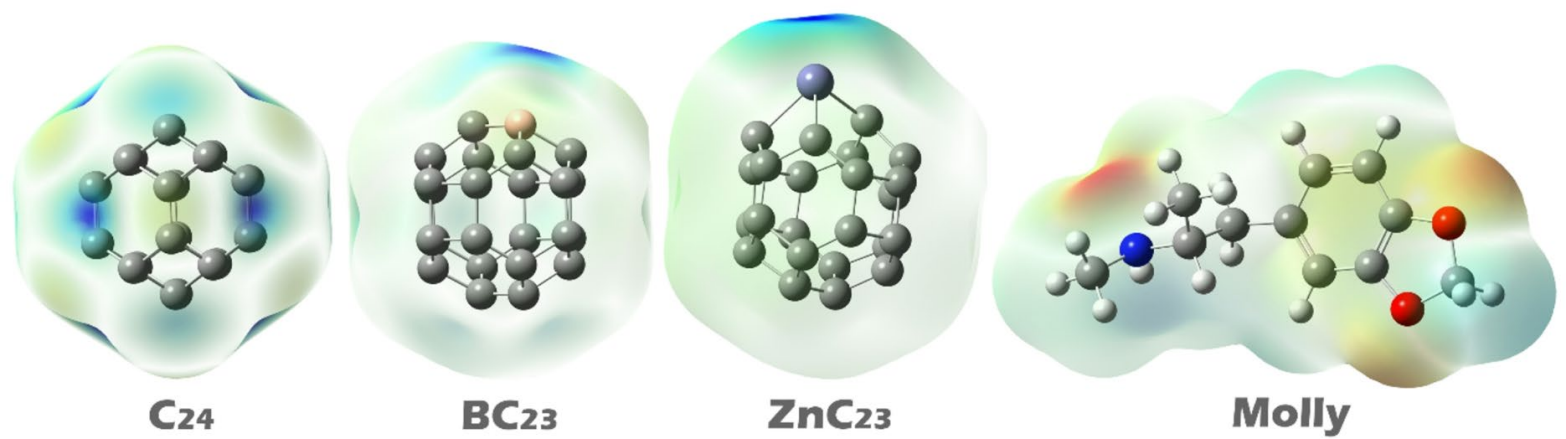
MEP contours for each of the structures studied in this work.

In the MEP map of C_24_, the blue dye (indicating regions of positive electrostatic potential) is uniformly distributed around the carbon atoms, suggesting a relatively even charge distribution across the molecule. In contrast, in the doped structures BC_23_ and ZnC_23_, the blue dye is concentrated on the boron and zinc atoms, respectively, highlighting these dopant sites as localized centers of positive potential. In the drug molecule Molly, the red dye (representing regions of high negative electrostatic potential) is prominently located on the nitrogen and oxygen atoms, which are electron-rich and likely to interact with positively charged or electron-deficient regions of other molecules (See Fig. 3).

Based on this analysis, the best interaction points for complex formation can be identified. For pristine C_24_, due to its uniform positive potential, interaction with Molly is expected to occur through general π–π or electrostatic interactions between carbon atoms with nitrogen or oxygen atoms of Molly. In BC_23_ and ZnC_23_, the interaction is expected to be more localized, with the boron and zinc atoms serving as active sites that directly interact with the nitrogen and oxygen atoms of Molly through electrostatic attraction.

**Fig. 4:**
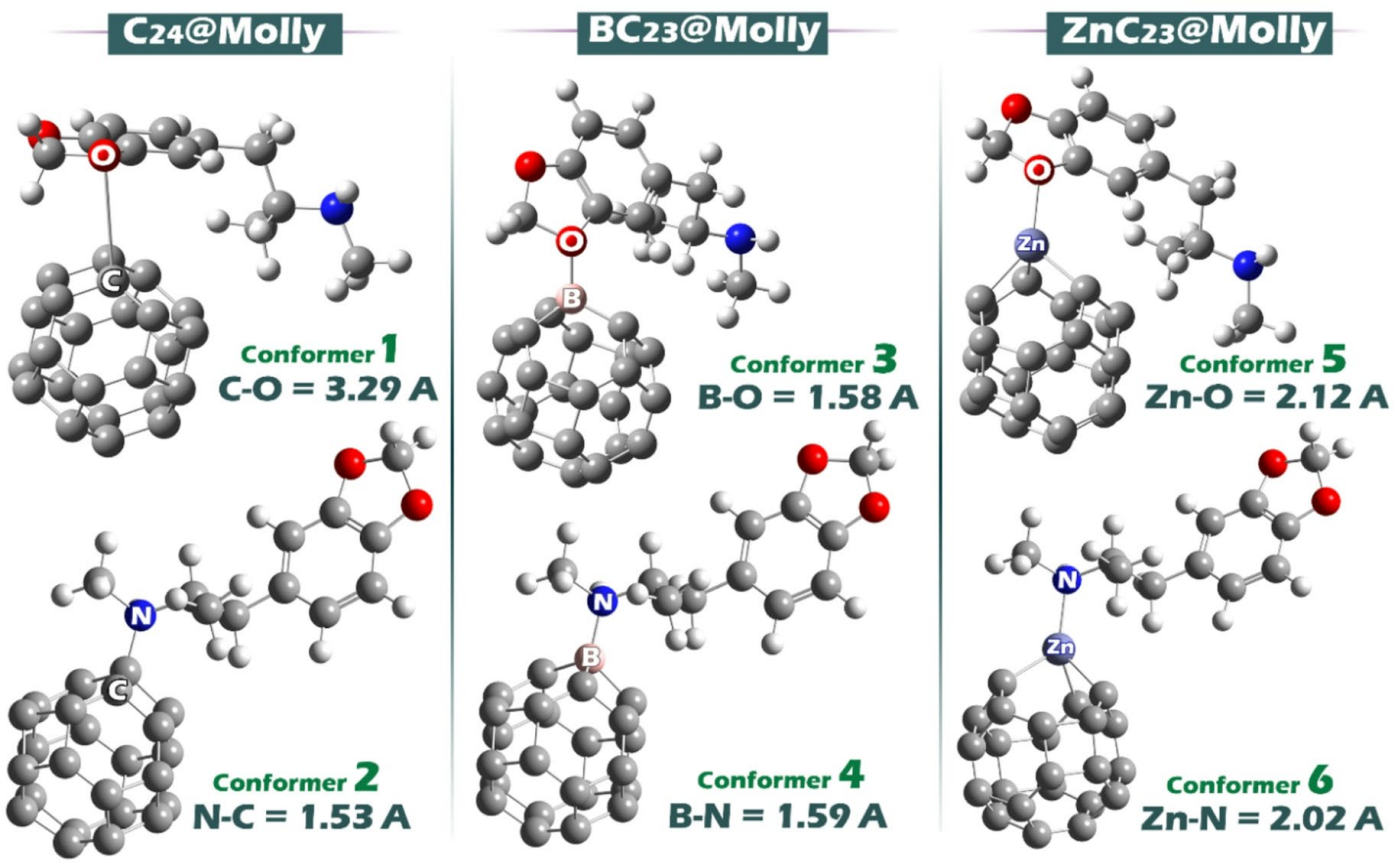
Optimized geometry of each of the designed complexes.

Based on the MEP findings, the expected molecular complexes between Molly and each of the C_24_, BC_23_, and ZnC_23_ structures were designed. The optimized geometries of the resulting complexes are shown in Fig. 4.

The data in the table show the bond lengths formed between the drug molecule Molly and the sensor structures C_24_, BC_23_, and ZnC_23_ in different conformers. In the C_24_@Molly complex, the C–O bond in Conformer 1 is relatively long at 3.29 Å, indicating a weak interaction, while the C–N bond in Conformer 2 is significantly shorter at 1.53 Å, suggesting a stronger interaction. In the BC23@Molly complex, both the B–O and B–N bonds in Conformers 3 and 4 are around 1.58–1.59 Å, indicating strong and nearly equal interactions with both oxygen and nitrogen atoms of Molly. In the ZnC_23_@Molly complex, the Zn–O and Zn–N bonds are 2.12 Å and 2.02 Å, respectively. These values are longer than the B–X bonds but still indicate relatively strong interactions, especially compared to the C–O bond in C_24_. Overall, the bond lengths suggest that doping with boron or zinc enhances the interaction strength between the sensor structure and Molly compared to the undoped C_24_.

### Energy of HOMO/LUMO orbitals and quantum parameters

In the design of sensors and the study of molecular complexes, examining parameters such as the energy gap (HLG), chemical softness (S), chemical hardness (η), chemical potential (µ), maximum charge transferred (ΔNmax), and charge transfer based on electrophilicity (ECT) is essential for understanding reactivity and stability. The energy gap (HLG) between the HOMO and LUMO levels provides insight into the electronic conductivity and chemical reactivity of the sensor; a smaller gap typically indicates higher reactivity and better sensing ability^51^. Chemical softness (S) and hardness (η) describe a molecule’s tendency to donate or accept electrons (soft systems are more reactive and adaptable for sensing, while harder systems are more stable)^52^. Chemical potential (µ) reflects the molecule’s tendency to gain or lose electrons, helping to predict the direction of electron flow during interaction^53^. The maximum charge transferred (ΔNmax) quantifies how much charge can be exchanged between the sensor and the analyte, which is critical for signal generation^54^. Also, ECT (electrophilicity-based charge transfer) helps to assess the nature and direction of charge transfer in the complex^55^. Together, these parameters provide a comprehensive picture of the sensor’s electronic properties and its efficiency in detecting target molecules.

**Table 2:** Energy values ​​of orbitals HOMO (eV) and LUMO (eV) as well as HLG (eV), (eV), S (eV^−1^), (eV), ∆N_max_ and ECT for each of the conformers studied in this work.

Structure	Conformer	LUMO	HOMO	HLG	η	S	µ	∆N_max_	ECT
Sensor									
C_24_	–	− 1.61	− 7.73	6.12	3.06	0.16	− 4.67	1.52	–
BC_23_	–	− 1.99	− 7.67	5.68	2.84	0.17	− 4.83	1.70	–
ZnC_23_	–	− 1.78	− 7.02	5.24	2.62	0.19	− 4.40	1.67	–
Complex/conformer									
C_24_@Molly	1	− 1.60	− 7.52	5.92	2.94	0.17	− 4.56	1.55	0.03
	2	− 0.93	− 6.69	5.76	2.88	0.17	− 3.81	1.32	− 0.20
BC_23_@ Molly	3	− 1.26	− 7.27	6.01	3.01	0.16	− 4.26	1.41	− 0.29
	4	− 1.03	− 7.11	6.08	3.04	0.16	− 4.07	1.33	− 0.37
ZnC_23_@ Molly	5	− 1.79	− 7.01	5.22	2.61	0.19	− 4.40	1.68	0.01
	6	− 1.65	− 6.92	5.27	2.63	0.19	− 4.28	1.62	− 0.05

The data in Table 2 reveal important trends in the electronic and chemical properties of the sensor structures (C_24_, BC_23_, and ZnC_24_) and their corresponding complexes with the drug molecule Molly. Comparing the pristine structures first, C24 has the highest energy gap (HLG) of 6.12 eV, indicating lower π-electron mobility and chemical reactivity. Doping with boron and zinc reduces the HLG to 5.68 eV in BC_23_ and 5.24 eV in3, suggesting enhanced π-electron delocalization and greater reactivity. Correspondingly, the chemical hardness (η) decreases from 3.06 eV in C_24_ to 2.84 eV in BC_23_ and 2.62 eV in ZnC_23_, while the chemical softness (S) increases from 0.16 to 0.19 eV^− 1^, reinforcing the increased electronic responsiveness of the doped structures. The chemical potential (µ) also varies, with BC_23_ being slightly more negative (− 4.83 eV), indicating a stronger tendency to accept electrons, and ZnC23 being less negative (− 4.40 eV), suggesting a higher tendency to donate electrons. The maximum charge transferred (ΔNmax) follows a similar trend, increasing from 1.52 in C24 to 1.70 in BC_23_ and 1.67 in ZnC_23_, further supporting the greater charge accommodation capacity of the doped systems.

Upon complexation with Molly, the properties of each structure shift in response to the interaction. In the C_24_@Molly complex, Conformer 1 shows a slight decrease in HLG to 5.92 eV, while Conformer 2 shows a more pronounced reduction to 5.76 eV, indicating increased π-electron mobility and interaction in Conformer 2. The chemical potential increases in both conformers (− 4.56 and − 3.81 eV), reflecting a greater tendency of the complex to donate electrons. The ΔNmax values remain relatively high (1.55 and 1.32), indicating sustained charge transfer capacity. The ECT values for C_24_@Molly reveal a change in charge transfer direction: Conformer 1 has a positive ECT (0.03), indicating no net electron transfer from the sensor to Molly, while Conformer 2 has a negative ECT (− 0.20), meaning that electrons are transferred from Molly to the sensor.

In the BC_23_@Molly complex, both conformers exhibit slightly increased HLG values (6.01 and 6.08 eV), suggesting a marginal decrease in π-electron mobility compared to the pristine BC_23_ structure. The chemical potential becomes less negative (− 4.26 and − 4.07 eV), indicating reduced electron-accepting strength, and ΔNmax values decrease to 1.41 and 1.33, pointing to a slight reduction in charge transfer capability. However, both conformers exhibit negative ECT values − 0.29 and − 0.37), which clearly indicates that Molly donates electrons to the sensor in both cases, and BC_23_ acts as an electron acceptor, likely due to the presence of the electron-deficient boron atom.

In the ZnC_23_@Molly complex, the HLG values (5.22 and 5.27 eV) remain almost unchanged from the pristine structure, showing that complex formation does not significantly affect the π-electron behavior. The chemical potential shifts slightly to − 4.40 and − 4.28 eV, and ΔNmax values remain high (1.68 and 1.62), indicating that the charge transfer capacity is largely preserved. The ECT values differ between the two conformers: Conformer 5 shows a slightly positive value (0.01), suggesting no net charge transfer from the sensor to Molly, while Conformer 6 shows a negative ECT (− 0.05), indicating that Molly acts as the electron donor in that configuration.

Based on the comprehensive analysis of the electronic properties, charge transfer behavior, and interaction strength between the sensor structures (C_24_, BC_23_, and ZnC_23_) and the drug molecule Molly, BC23 emerges as the most promising structure for use as a Molly sensor.

**Fig. 5:**
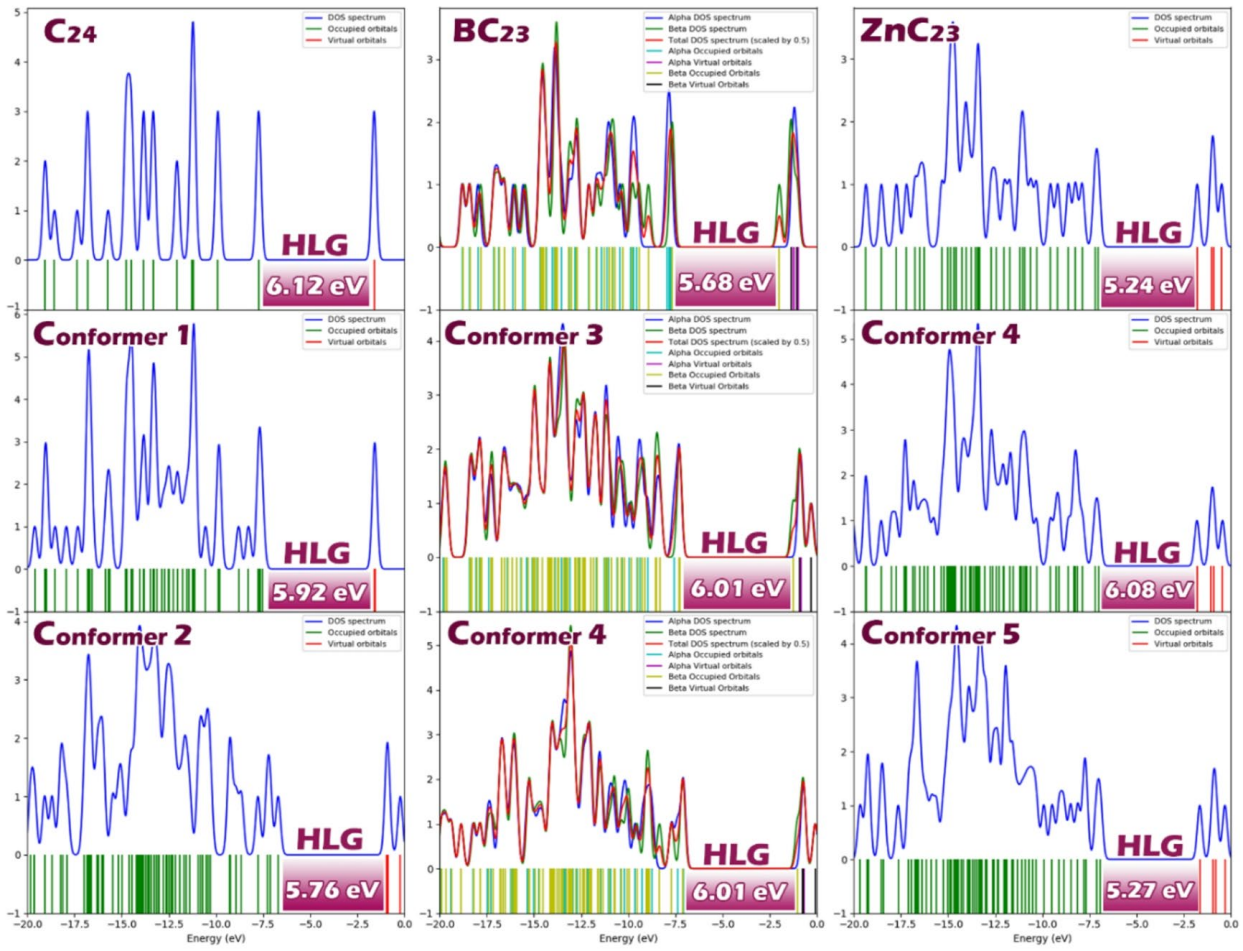
Display of HLG values using DOS plots for each of the structures studied in this work.

Density of States (DOS) plots are an excellent tool for visualizing the distribution of electronic energy levels and identifying the energy gap (HLG) between the highest occupied and lowest unoccupied molecular orbitals. They provide a clear graphical representation of the electronic structure, making it easier to interpret the reactivity and conductivity of a system^56,57^. For this purpose, the DOS plots for each of the designed Sensor-Molly complexes are shown in Fig. 5. These plots visually confirm the energy gap values reported in Table 2, supporting the numerical analysis and highlighting the effects of doping and complexation on the electronic properties of the sensors.

Representing the spatial shape of molecular orbitals, particularly the HOMO and LUMO, is essential for understanding how and where electronic interactions occur within a complex. Visualizing the location of these orbitals helps determine which parts of the molecule are actively involved in charge transfer, chemical reactivity, or sensor-analyte interaction. Specifically, the HOMO position represents the region that donates electrons, while the LUMO represents the region that accepts electrons^58,59^.

**Fig. 6:**
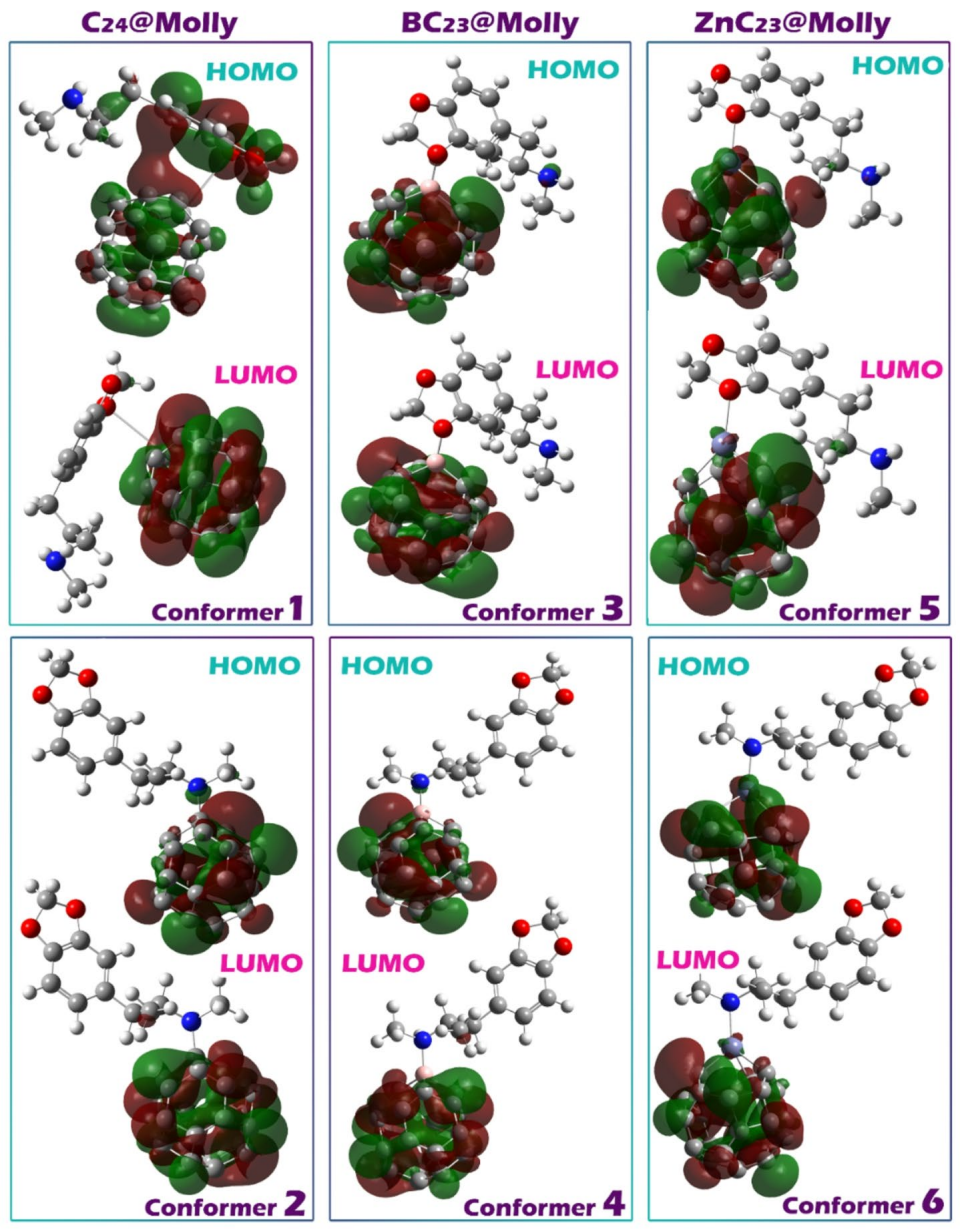
Distribution of HOMO and LUMO orbitals in each of the sensor-molly complexes.

In the studied complexes, visual analysis reveals that both the HOMO and LUMO orbitals are localized entirely on the sensor structures rather than on the drug Molly (Fig. 6). This spatial distribution indicates that the sensor is the primary site for both electron donation and acceptance during interaction. Such a feature is highly desirable in sensor design, as it means the sensor can independently mediate the electronic response upon binding with the analyte, enhancing the stability and reliability of signal generation without relying on electronic contributions from the target molecule.

### Dipole moment

The dipole moment is a key parameter in sensor design because it reflects the distribution of electrical charge within a molecule. When a sensor binds to a drug molecule, any change in dipole moment can signal a shift in electron density, which is critical for generating an electrical response. This change forms the basis for detection in many electronic sensors, as it can be measured as a change in voltage or current^60,61^. Additionally, the dipole moment provides insight into the solubility of sensor-drug complexes, especially in polar environments. Molecules with higher dipole moments typically dissolve more easily in polar solvents, which is important for ensuring the effective dispersion and functionality of the sensor in real-world applications^62^. For this purpose, the dipole moment was calculated for each of the designed structures in the presence and absence of Molly, and the results were reported in Table 3.

**Table 3:** Dipole moment values in each of the instruments designed in this work.

Structure	Conformer	Dipole moment
C_24_	–	0.0
BC_23_	–	0.24
ZnC_23_	–	6.09
C_24_@Molly	1	1.30
	2	15.68
BC_23_@ Molly	3	7.52
	4	12.31
ZnC_23_@ Molly	5	6.26
	6	11.17

The data presented in Table 3 highlight the impact of Molly binding and elemental doping on the dipole moment of the sensor structures. Initially, the undoped C_24_ structure exhibits a dipole moment of 0.0 Debye, indicating a highly symmetric and nonpolar distribution of charge. However, upon binding with Molly, the dipole moment increases significantly to 1.30 Debye in Conformer 1 and 15.68 Debye in Conformer 2. This change suggests a substantial redistribution of electron density upon complex formation, which is favorable for generating an electrical signal in sensing applications.

The BC_23_ sensor, doped with boron, shows a slight intrinsic dipole moment of 0.24 Debye, reflecting a minor break in symmetry due to doping. When complexed with Molly, its dipole moment increases markedly to 7.52 and 12.31 Debye in Conformers 3 and 4, respectively. This increase confirms the formation of polar complexes and the potential of BC_23_ to act as an effective sensor through dipole-induced signal generation.

ZnC_23_, which is doped with zinc, exhibits a much higher dipole moment in its unbound state (6.09 Debye), suggesting strong intrinsic polarity due to the presence of the metal atom. Upon interaction with Molly, the dipole moment shows further elevation to 6.26 and 11.17 Debye for Conformers 5 and 6, respectively. These values indicate that ZnC_23_ maintains and even enhances its polarity after complexation, supporting its solubility and signal generation capability.

Overall, the data clearly show that doping significantly increases the initial dipole moment of the sensor structures and enhances the sensitivity of the sensors to Molly through greater dipole moment shifts upon binding. These changes are crucial for both effective solubility and reliable electronic signal generation in real-world sensor applications.

### Adsorption energy, recovery time and electrical conductivity

In sensor design, considering adsorption energy, recovery time, and electrical conductivity is essential for evaluating performance and efficiency. Adsorption energy indicates how strongly the analyte binds to the sensor surface; moderate values are preferred to ensure stable interaction without making desorption too difficult^63^. Recovery time reflects how quickly the sensor returns to its original state after detecting the analyte, which is crucial for real-time and repeatable sensing^64^. Electrical conductivity plays a key role in signal transmission; higher conductivity enhances the sensor’s ability to detect even small changes in the presence of the analyte^65^. For this purpose, each of these parameters was calculated, and the results were reported in Table 4.

**Table 4:** Adsorption energy (E_ads_), electrical conductivity (σ) and recovery time (τ) values ​​in each of the studied structures.

Structure	Conformer	E_ads_ (kcal mol^− 1^)	$\:{\tau\:}$ (s)	($\:{\sigma\:}$) (A m^− 2^)
C_24_	–	–	–	2.73 × 10^9^
NC_23_	–	–	–	2.76 × 10^9^
ZnC_23_	–	–	–	2.78 × 10^9^
C_24_@Molly	1	− 12.50	1.45 × 10^− 4^	2.75 × 10^9^
	2	− 14.43	3.8 × 10^− 4^	2.75 × 10^9^
NC_23_@Molly	3	− 15.06	1.1 × 10^− 3^	2.74 × 10^9^
	4	− 18.19	2.2 × 10^− 1^	2.74 × 10^9^
ZnC_23_@Molly	5	− 13.80	1.2 × 10^− 4^	2.78 × 10^9^
	6	− 14.43	3.8 × 10^− 4^	2.78 × 10^9^

The propensity for conformer formation within each complex is fundamentally governed by the adsorption energy (Eads), which reflects the strength of interaction between the sensor surface and the Molly molecule. A more negative adsorption energy corresponds to a stronger binding affinity and thus a higher likelihood of stable complex formation.

In the case of the C_23_@Molly complex, two conformers were analyzed: conformer 1 exhibits an adsorption energy of − 12.50 kcal mol^− 1^, whereas conformer 2 displays a more negative value of − 14.43 kcal mol^− 1^. This indicates that conformer 2 interacts more strongly with Molly and is therefore thermodynamically favored, making it the more probable structure within this system.

Similarly, for the BC23@Molly complex, conformer 3 shows an adsorption energy of − 15.06 kcal.mol^− 1^, while conformer 4 possesses a substantially more negative value of − 18.19 kcal mol^− 1^. The pronounced difference suggests that conformer 4 has a significantly higher binding affinity for Molly, thereby representing the preferred and more stable conformer.

In the ZnC_23_@Molly system, conformer 5 and conformer 6 exhibit adsorption energies of − 13.80 kcal.mol^− 1^ and − 14.43 kcal mol^− 1^, respectively. Although the difference is modest, conformer 6 demonstrates a stronger interaction and is thus more likely to form.

Consequently, based on adsorption energies alone, conformer 2 is favored in C_24_@Molly, conformer 4 in BC_23_@Molly, and conformer 6 in ZnC_23_@Molly, as these represent the most thermodynamically stable configurations within their respective complexes.

Building upon the identification of these most stable conformers, their functional performance was further assessed through recovery time (τ) and electrical conductivity (σ) to determine the most effective electrochemical sensor and the optimal adsorbent for Molly detection. Recovery time, indicative of the sensor’s ability to revert to its initial state post-detection, is critical for real-time and repeatable sensing; shorter recovery times are therefore desirable. Among the selected conformers, conformers 2 (C_24_@Molly) and 6 (ZnC_23_@Molly) both exhibit rapid recovery times of 3.8 × 10^− 4^ s, whereas conformer 4 (BC_23_@Molly) shows a considerably prolonged recovery time of 2.2 × 10^− 1^ s. This disparity suggests that although conformer 4 possesses strong adsorption, its slow recovery diminishes its suitability for dynamic sensing applications.

Regarding electrical conductivity, which directly influences the sensor’s signal transduction efficiency, conformer 6 (ZnC_23_@Molly) attains the highest conductivity value of 2.78 × 10^9^ A m^− 2^, closely followed by conformer 2 (C_24_@Molly) at 2.75 × 10^9^ A m^− 2^, and conformer 4 (BC_23_@Molly) at 2.74 × 10^9^ A m^− 2^. While the differences are subtle, the superior conductivity of conformer 6 enhances its potential for sensitive and rapid electrochemical response.

Integrating these parameters, conformer 6 (ZnC_23_@Molly) emerges as the optimal electrochemical sensor candidate, exhibiting a balanced profile of strong adsorption (− 14.43 kcal mol^− 1^), fast recovery, and highest conductivity, which collectively ensure effective detection and signal transduction. Conversely, for applications prioritizing adsorption capacity and molecular capture, where strong and stable binding is paramount despite slower reversibility, conformer 4 (BC_23_@Molly) is identified as the best adsorbent. Its highly negative adsorption energy (− 18.19 kcal mol^− 1^) underscores its potential for robust Molly retention in passive or filtration-based systems.

### UV spectrum

**Fig. 7:**
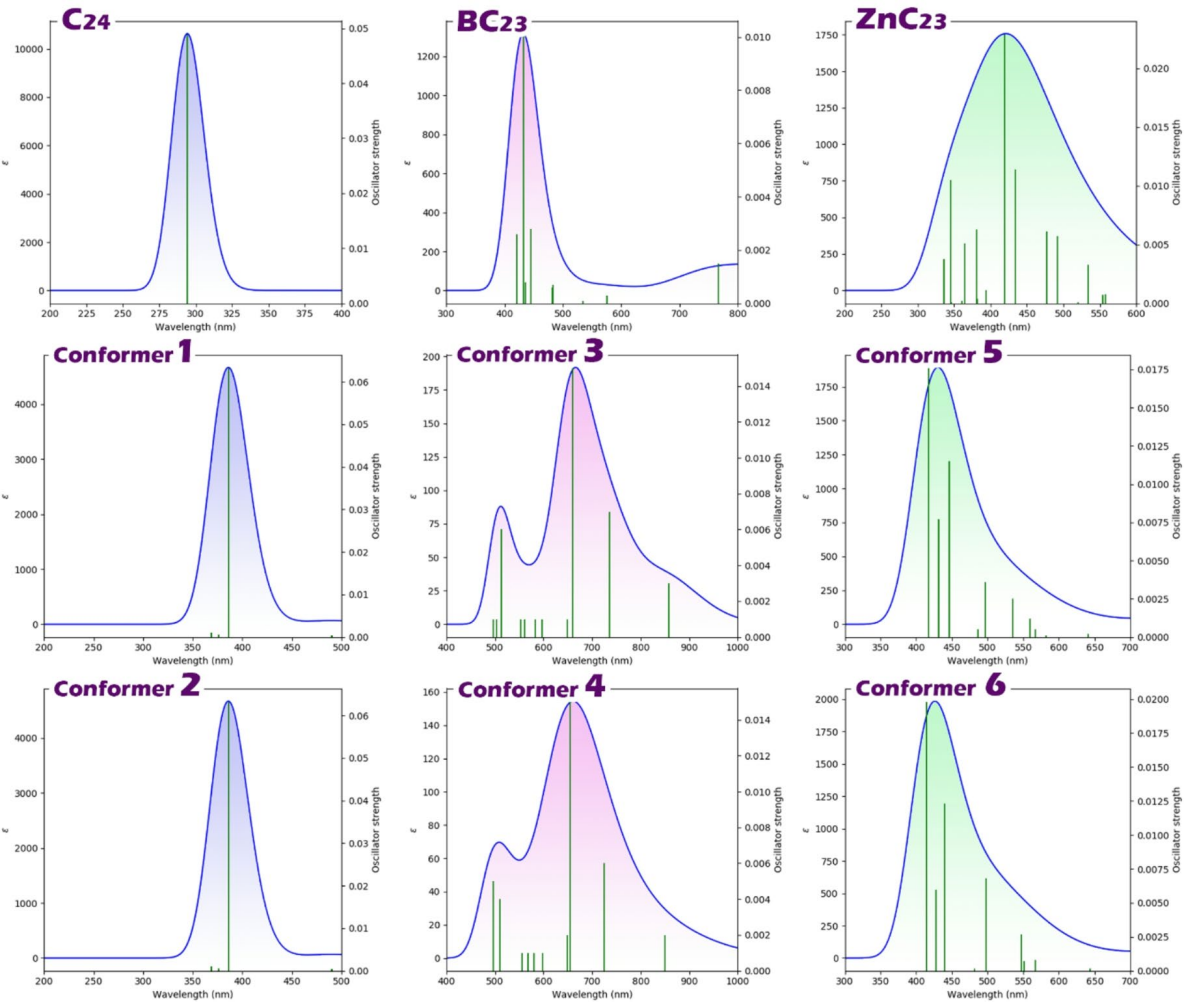
UV spectra for C_24_, BC_23_, and ZnC_23_ in the presence/absence of molly.

In the design of colorimetric sensors, the parameters λmax, excitation energy (Ex), and oscillator strength (ƒ) are fundamental for evaluating sensor performance. The wavelength of maximum absorption (λmax) determines the specific color change the sensor exhibits upon analyte interaction, which is crucial for visual detection^66^. Excitation energy (Ex) corresponds to the energy required to promote an electron from the ground state to an excited state; lower Ex values typically indicate easier electronic transitions, enhancing sensor sensitivity^67^. Oscillator strength (ƒ) reflects the probability and intensity of the electronic transition; higher ƒ values correspond to stronger absorption and more vivid color changes, improving the sensor’s responsiveness and detectability^68^. Each of these parameters was calculated, and the results were reported in Table 5; Fig. 7.

**Table 5:** Values of $\lambda$max, Ex and ƒ for each of the C24, BC23 and ZnC23 sensors in the presence/absence of molly.

Structure	Conformer	λmax (nm)	Eex (eV)	ƒ
C_24_	–	294	4.21	0.048
BC_23_	–	444	2.79	0.028
ZnC_23_	–	419	2.95	0.022
C_24_@Molly	1	386	3.21	0.063
	2	461	2.68	0.007
BC_23_@Molly	3	660	1.87	0.015
	4	654	1.89	0.015
ZnC_23_@Molly	5	417	2.97	0.017
	6	414	2.99	0.019

The comparative analysis of the optical parameters (maximum absorption wavelength (λmax), excitation energy (Eex), and oscillator strength (ƒ)) for the three studied sensor systems (C_24_, BC_23_, and ZnC_23_) in the presence and absence of Molly reveals significant differences in their colorimetric sensing potential. These parameters are critical for evaluating the optical response of a sensor, as λmax indicates the observed color, Eex reflects the energy required for electronic transitions, and ƒ corresponds to the intensity of light absorption, directly influencing the visibility of color change.

In the case of C_24_, the pristine sensor displays a λmax of 294 nm, Eex of 4.21 eV, and ƒ of 0.048. Upon interaction with Molly, two conformers are formed. Conformer 1 shows a redshift to 386 nm and a reduced excitation energy of 3.21 eV, accompanied by an increased oscillator strength of 0.063, indicating a stronger and more visible absorption. Conformer 2 exhibits an even greater redshift to 461 nm and lower Eex of 2.68 eV; however, the oscillator strength drops sharply to 0.007, suggesting a weak and possibly less detectable optical response. Therefore, conformer 1 represents a more effective optical response for C_24_@Molly due to its balanced redshift and enhanced absorption intensity.

For BC_23_, the initial λmax is 444 nm, with an excitation energy of 2.79 eV and an ƒ value of 0.028. Upon Molly adsorption, both conformers 3 and 4 exhibit significant redshifts to approximately 660 nm, with excitation energies decreasing to 1.87 and 1.89 eV, respectively. Although the oscillator strength slightly decreases to 0.015, the large shift in λmax to the visible red region indicates a highly distinguishable and efficient optical response. The strong redshift reflects effective charge transfer interactions, likely facilitated by π→π* or n→π* transitions, which are dominant in conjugated and heteroatom-containing systems and are typically associated with high second-order perturbation energies (E^2^), indicating strong donor–acceptor stabilization.

In contrast, ZnC23 shows a relatively stable optical profile. The pristine form has a λmax of 419 nm, Eex of 2.95 eV, and ƒ of 0.022. Upon interaction with Molly, the conformers display only minimal shifts in λmax (417 and 414 nm) and negligible changes in excitation energy and oscillator strength. This indicates a weak interaction with Molly and an insufficient optical response, making ZnC23 a poor candidate for colorimetric sensing in this application.

Overall, the dominance of π→π* transitions, which are more responsive to molecular interactions and result in stronger color changes, is evident in the systems that show significant λmax shifts and favorable Eex and ƒ values. These transitions are enhanced by effective electron delocalization and charge transfer, as reflected in NBO interactions and second-order perturbation stabilization energies.

According to the results obtained, among the three systems, BC_23_ shows the most pronounced and visually observable redshift upon binding with Molly, combined with sufficiently low excitation energy and moderate oscillator strength. These features make BC_23_ the most effective colorimetric sensor for Molly detection, providing clear spectral shifts and strong visual contrast that are essential for practical sensor applications.

### Natural bond orbitals (NBO)

NBO analysis provides insights into the distribution of electrons in bonding and antibonding orbitals and identifies donor-acceptor interactions within the molecule^69^. The second-order perturbation energy (E^2^) quantifies the stabilization resulting from these interactions, with higher E^2^ values indicating stronger electronic coupling and more effective charge transfer. This enhanced interaction often correlates with increased sensor sensitivity and a more distinct colorimetric response^70,71^. The value of the second-order perturbation energy matrix (E^2^) using the Eq. (11):

11 $$\:{{E}}^{2}={\varDelta\:{E}}_{{i},{j}}-{q}\frac{{{F}}^{2}({i},{j})}{{{E}}_{{j}}-{{E}}_{{i}}}$$

Electron transitions relevant to colorimetric sensing include σ→σ*, π→π*, lone pair (LP)→σ*, and LP→π* transitions, all of which can be related to the donor-acceptor interactions revealed by NBO analysis and quantified by E^2^ values. Among these, π→π* transitions are typically dominant due to their involvement in conjugated systems, which facilitate strong light absorption and efficient electron excitation. These transitions usually have higher oscillator strengths, resulting in pronounced changes in color upon analyte binding, making them central to the sensing mechanism^72–74^. In contrast, σ→σ* and LP-involved transitions tend to contribute less intensely to optical changes. Therefore, analyzing NBO interactions and maximizing E^2^ values (particularly those associated with π→π* transitions) are essential strategies for optimizing the sensitivity and selectivity of colorimetric sensors.

**Table 6:** Calculated values ​​for the second-order perturbation energy matrix (E2) using NBO analysis in each of the conformers studied in this work.

Complex	Conformer	Donor (i)	Type	Acceptor (j)	Type	E^(2)^ kcal mol^− 1^	E(j)-E(i) a.u.	F(i, j) a.u.
C_24_@Molly	1	$\:\sigma\:$	C1-C2	$\:{\sigma\:}^{*}$	C1-C3	2.09	1.20	0.045
		$\:\pi\:$	C1-C12	$\:{\pi\:}^{*}$	C9-C10	15.83	0.34	0.069
		LP (1)	N32	$\:{\pi\:}^{*}$	C30-C31	5.33	0.36	0.039
	2	$\:\sigma\:$	C1-C2	$\:{\sigma\:}^{*}$	C2-C14	2.90	1.38	0.057
		$\:\pi\:$	C1-C15	$\:{\pi\:}^{*}$	C13-C16	17.60	0.45	0.100
		LP (1)	C8	$\:{\pi\:}^{*}$	C20-C22	0.84	0.27	0.015
BC_23_@Molly	3	$\:\sigma\:$	C1-C2	$\:{\sigma\:}^{*}$	C3-C13	2.53	1.15	0.068
		$\:\pi\:$	C6-C7	$\:{\pi\:}^{*}$	C20-C22	20.62	0.46	0.124
		LP (1)	C8	$\:{\pi\:}^{*}$	C26-O21	0.04	0.39	0.006
	4	$\:\sigma\:$	C1-C2	$\:{\sigma\:}^{*}$	C2-C13	1.00	1.37	0.047
		$\:\pi\:$	C3-C23	$\:{\pi\:}^{*}$	C16-C17	21.13	0.46	0.125
		LP (1)	C8	$\:{\pi\:}^{*}$	C3-C23	55.97	0.28	0.173
ZnC_23_@Molly	5	$\:\sigma\:$	C1-C2	$\:{\sigma\:}^{*}$	C5-C4	4.13	0.81	0.054
		$\:\pi\:$	C6-C7	$\:{\pi\:}^{*}$	C20-C22	11.65	0.44	0.065
		LP (1)	C4	$\:{\pi\:}^{*}$	C2-C13	4.43	0.41	0.039
	6	$\:\sigma\:$	C1-C2	$\:{\sigma\:}^{*}$	C1-C14	2.94	1.35	0.056
		$\:\pi\:$	C1-C14	$\:{\pi\:}^{*}$	C12-C15	12.84	0.42	0.066
		LP (1)	C4	$\:{\pi\:}^{*}$	C2-C13	3.83	0.42	0.037

Based on the data presented in Table 6 and considering the principles outlined through NBO analysis and second-order perturbation energy matrix (E^2^), a detailed evaluation of the electronic transitions and their contributions to the performance of the colorimetric sensors can be made.

In C_24_@Molly, conformer 1 shows a significant π→π* transition from C1–C12 to C9–C10 with an E^2^ value of 15.83 kcal mol^− 1^, suggesting a strong conjugative interaction contributing to effective electron delocalization and optical activity. This transition dominates over the σ→σ* (2.09 kcal mol^− 1^) and LP→π* (5.33 kcal mol⁻¹) interactions. In conformer 2, the π→π* interaction is even stronger (17.60 kcal mol⁻¹ between C1–C15 and C13–C16), accompanied by a higher F(i, j) value (0.100), indicating an even more efficient donor–acceptor overlap. The LP→π* interaction in this case is minor (0.84 kcal.mol^− 1^), suggesting that the π→π* pathway plays the primary role in modulating the sensor’s optical response.

In BC_23_@Molly, the π→π* interactions are particularly pronounced. Conformer 3 exhibits a π→π* interaction from C6–C7 to C20–C22 with an E^2^ value of 20.62 kcal mol^− 1^ and a strong F(i, j) of 0.124, while the LP→π* interaction is negligible (0.04 kcal mol^− 1^), indicating minimal involvement of lone pair electrons. Notably, in conformer 4, a remarkable LP→π* interaction is observed (55.97 kcal mol^− 1^ from C8 to C3–C23), the highest among all conformers, coupled with a substantial π→π* contribution (21.13 kcal mol^− 1^). The calculated second-order disorder energy matrix (E^2^) values obtained from NBO analysis represent the stabilization energies arising from donor–acceptor interactions between the molecular orbitals of the sensor and the Molly molecule. These values quantitatively describe the intermolecular charge transfer interactions (primarily π→π* and LP→π* transitions) that contribute to the overall stability of the complexes. The higher E^2^ values observed in BC23@Molly conformer 4 highlight the predominance of π→π* coupling and lone pair donation from the analyte toward the fullerene π*-orbitals, confirming that these intermolecular charge transfer interactions are the dominant contributors to sensor–analyte binding and the resulting optical response.

For ZnC_23_@Molly, both conformers show moderately strong π→π* interactions. Conformer 5 exhibits a π→π* transition with an E^2^ of 11.65 kcal mol^− 1^ (C6–C7 → C20–C22), while conformer 6 displays a slightly higher value of 12.84 kcal mol^− 1^ (C1–C14 → C12–C15). These interactions dominate over the LP→π* contributions (4.43 and 3.83 kcal mol^− 1^, respectively), reaffirming the primacy of π→π* transitions in optical modulation. The σ→σ* interactions are moderate in both cases (4.13 and 2.94 kcal mol⁻¹), but their impact on optical activity is less significant than the π→π* transitions.

Among all, BC_23_@Molly conformer 4 stands out with exceptionally high E^2^ values for both π→π* and LP→π* transitions, suggesting an intensely stabilized electronic system with high potential for pronounced and responsive color change. Therefore, BC_23_ is the most promising candidate for colorimetric detection of Molly, due to its optimal electron donor-acceptor interactions and potential for a strong chromatic shift upon analyte binding.

### Non-covalent interaction (NCI)

NCI analysis plots, which map the reduced density gradient (RDG) against sign(λ_2_)ρ, are crucial for visualizing and identifying non-covalent interactions within sensor-drug complexes^75^. These plots help differentiate between attractive interactions (e.g., hydrogen bonding), weak van der Waals forces, and repulsive steric effects^76,77^. By revealing the strength and nature of these interactions, NCI analysis supports the rational design of colorimetric and electrochemical sensors with improved selectivity, stability, and sensitivity toward target molecules.

**Fig. 8:**
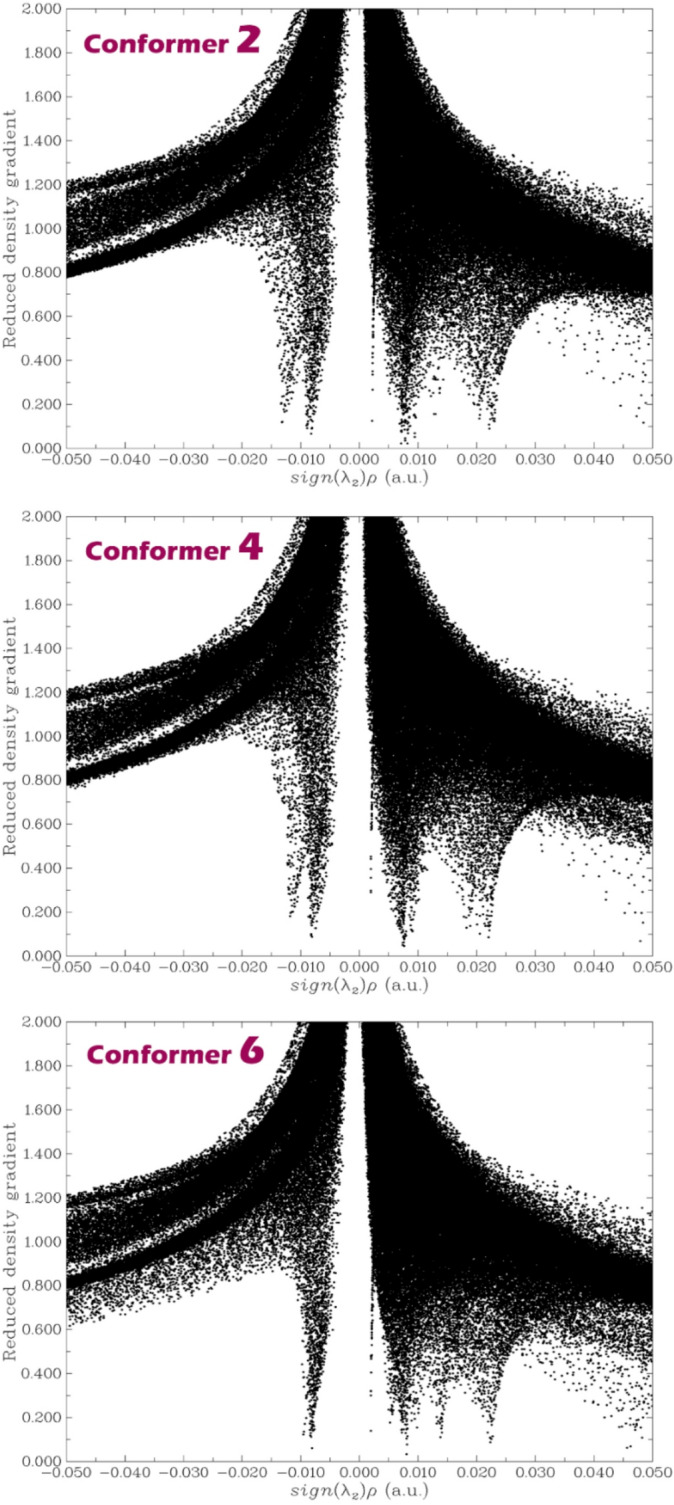
NCI contours for conformers 2,4 and 6.

The NCI (Non-Covalent Interaction) contours of conformers 2, 4, and 6 were studied in detail to better understand the nature and distribution of weak interactions that may contribute to their favorable adsorption energies. These conformers were selected based on their lower (more negative) adsorption energy values, which suggest stronger and more stable interaction with the adsorbent surface or host system (Fig. 8). In all three conformers, a prominent peak appears near sign(λ_2_)ρ ≈ 0, indicating the dominance of weak van der Waals interactions that are typically associated with dispersion forces. These interactions play a fundamental role in stabilizing adsorbed complexes through broad, non-directional contact areas. Notably, Conformer 2 exhibits a relatively wider and denser region extending into negative values of sign(λ_2_)ρ, suggesting the presence of stronger attractive interactions such as hydrogen bonding or charge-assisted interactions. This characteristic correlates well with its highly favorable adsorption energy, indicating that directional, stabilizing non-covalent interactions significantly contribute to its overall binding strength.

Conformer 4 also shows notable interaction features. While similar to Conformer 2 in overall contour shape, it presents a slightly increased density of points toward positive sign(λ_2_)ρ values, implying the presence of steric repulsion or electron cloud overlap in some regions of the complex. Despite this, the extent of attraction remains substantial, supporting its strong adsorption energy and suggesting that the balance between stabilizing and destabilizing forces still results in effective adsorption.

Conformer 6 displays a well-balanced distribution with pronounced peaks on both sides of the central axis, indicating a coexistence of attractive and repulsive interactions. The symmetric nature of the plot and the relatively wide range of interaction zones suggest a complex but cooperative interaction profile. These features are consistent with its favorable adsorption energy and imply that while repulsive forces are present, they are effectively counterbalanced by a robust network of weak but stabilizing interactions, such as π–π stacking or dispersion contacts.

Overall, NCI analysis confirms that conformers 2, 4, and 6 exhibit richer and broader noncovalent interaction profiles in their RDG plots versus (λ_2_)ρ sign. This correlation highlights the importance of weak intermolecular forces in stabilizing adsorbed states and supports the conclusion that adsorption energy is closely linked to the nature and density of non-covalent interactions within each conformer.

### QTAIM analysis

The Quantum Theory of Atoms in Molecules (QTAIM) is a theoretical approach used to study chemical bonding by analyzing electron density within molecules^78^. At Bond Critical Points (BCPs), QTAIM provides key descriptors such as electron density (ρ(r)), its Laplacian (∇^2^ρ(r)), and energy density components (kinetic (G(r)) and potential (V(r))) which help classify interactions between atoms^79^. According to Rozas et al., the nature of sensor-analyte interactions can be identified using BCP data. If the total energy density (Hb = V(r) + G(r)) and ∇^2^ρ(r) are both negative, the interaction is a strong hydrogen bond, indicating a covalent-like and highly stable bond. If Hb is positive and ∇^2^ρ(r) is negative, the interaction is medium-strength, showing moderate electrostatic character. If both Hb and ∇^2^ρ(r) are positive, the interaction is weak and dispersive, resembling van der Waals forces^80,81^.

**Fig. 9:**
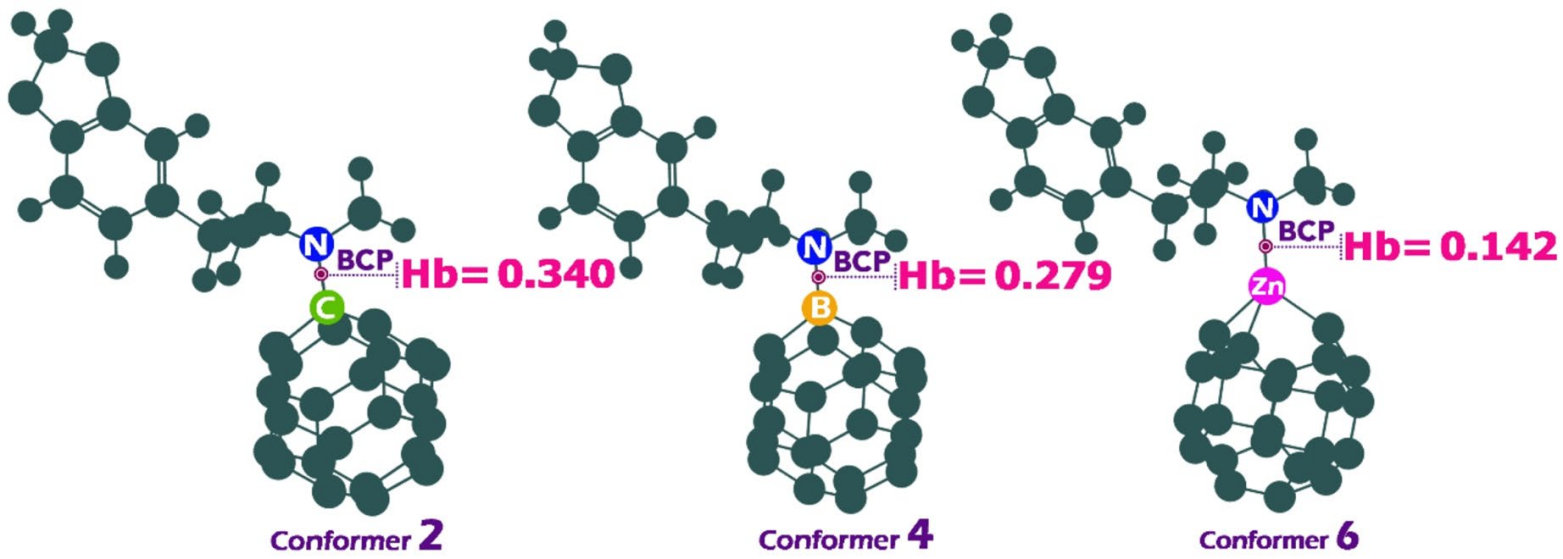
Value of the Hb in BCP for conformers 2,4, and 6.

**Table 7:** The obtained values for ρ(r), ∇^2^ρ(r), V(r), G(r), VIR, and the hb in the BCP.

Conformer	ρ(r)	∇^2^ρ(r)	V(r)	G(r)	VIR
2	0.226	0.134	0.237	0.103	0.341
4	0.132	− 0.092	0.093	0.185	0.279
6	0.087	− 0.066	0.037	0.104	0.142

Based on the QTAIM framework and the classification of interactions proposed by Rozas et al., the data for conformers 2, 4, and 6 can be interpreted by examining the electron density (ρ(r)), the Laplacian of electron density (∇^2^ρ(r)), and the total electron energy density (Hb = V(r) + G(r)) at the bond critical point (BCP). These parameters reveal the nature and strength of the non-covalent interactions involved in the adsorption process (Fig. 9; Table 7).

Conformer 2 displays the highest electron density at the BCP (ρ(r) = 0.226), a positive Laplacian value (∇^2^ρ(r) = 0.134), and a highly positive total energy density (Hb = 0.340). According to the classification, the combination of a positive ∇^2^ρ(r) and a positive Hb suggests that the interaction is weak in nature, dominated by dispersive van der Waals forces with limited electron sharing. Despite its high electron density, the positive Laplacian and energy density confirm that this interaction is not covalent-like but rather reflects electron depletion in the bonding region, typical of weak hydrogen bonds.

In contrast, Conformer 4 presents a significantly different profile. It has a lower electron density (ρ(r) = 0.132), but notably, both the Laplacian (∇^2^ρ(r) = − 0.092) and the total energy density (Hb = 0.279) show a change in character. The negative Laplacian indicates electron accumulation in the bonding region, suggesting a stabilizing interaction. However, the still-positive value of Hb suggests that the bond does not reach the strength of a covalent-like interaction. This points to a medium-strength hydrogen bond, where electrostatic attraction is more significant than in Conformer 2 and reflects a moderately stable complex.

Conformer 6 exhibits the lowest electron density (ρ(r) = 0.087) and also features a negative Laplacian (∇²ρ(r) = − 0.066), with a positive total energy density (Hb = 0.142). While the values are weaker than in Conformer 4, the negative Laplacian again suggests electron concentration between the interacting atoms. The moderate value of Hb, however, confirms that this interaction is also in the medium hydrogen bond category, though on the weaker end of the spectrum. These findings align with the observed adsorption behavior.

### Comparison with other literature

Compared with earlier studies, the present work demonstrates notable improvements in several key sensor performance indicators (excluding recovery time). Hosseinian et al. reported moderate adsorption energies for C_24_-drug systems and minimal optical shifts, whereas our boron-doped BC_23_@Molly complex achieved a significantly higher adsorption energy (− 18.19 kcal mol^−1^) and a strong λmax redshift from 444 nm to 660 nm, indicating markedly enhanced binding and optical sensitivity^82^. Similarly, Fouegue et al. found that B-doped C_24_ nanocages improved drug binding modestly; however, their reported HOMO-LUMO gaps remained above 6 eV, whereas our doped systems exhibited reduced gaps (BC_23_: 5.68 eV, ZnC_23_: 5.24 eV), enhancing electronic reactivity and signal responsiveness^83^.

In Swarna et al., hetero-nanocages interacting with hydroxyurea showed λmax shifts within the visible range but with limited oscillator strengths (ƒ < 0.015), while BC_23_@Molly in this work combined a large redshift with substantial donor-acceptor interactions (π→π* E^2^ = 21.13 kcal mol^−1^; LP→π* E^2^ = 55.97 kcal mol^−1^), producing a more intense and distinguishable optical response^84^. Mahani et al. used pristine C24 for pyridine derivatives and achieved modest changes in dipole moment upon adsorption, whereas our BC_23_ and ZnC_23_ complexes showed far greater dipole shifts (e.g., BC_23_@Molly up to 12.31 D), improving detectability in polar media^85^. Compared with Baei and Shojaei, who investigated toluene adsorption on fullerenes with moderate cohesive energies, our doped structures exhibited substantially higher cohesive energies (BC_23_: 194 kcal mol^−1^, ZnC_23_: 188 kcal mol^− 1^), indicating superior structural stability^86^.

In addition to the above, it is worth noting that while many studies have reported enhanced adsorption and stability in doped fullerene systems, other investigations have pointed out issues related to water insolubility, aggregation, and biocompatibility that can limit their direct analytical use. This highlights the importance of considering both the benefits and drawbacks of such nanocages when designing practical sensors^87,88^.

Overall, the integration of boron and zinc dopants in C_24_ significantly improved adsorption strength, cohesive stability, electronic reactivity, dipole responsiveness, and optical detectability relative to all referenced studies. These enhancements position BC_23_ as an exceptional colorimetric sensor and ZnC_23_ as a highly efficient electrochemical sensor, outperforming comparable fullerene-based systems reported to date.

### Future work

Selectivity is one of the most critical parameters in evaluating the performance of a sensor, as real-world samples often contain multiple coexisting compounds that may interfere with the detection of the target analyte. In the present study, while the primary focus was on assessing the intrinsic sensing capability of pristine and doped C_24_ fullerenes toward Molly, the effect of possible interferences is also relevant to ensure practical reliability. However, in practical environments, other substances with similar donor atoms (such as methamphetamine, caffeine, or ephedrine) may compete for the same adsorption sites, potentially influencing sensor response intensity. Although such competitive interactions were not directly simulated in this work, the observed electronic and structural properties of the doped systems imply that stronger, directional binding through hydrogen bonding and charge-assisted interactions could help distinguish Molly from related compounds. Future computational and experimental studies should therefore include interference modeling and cross-sensitivity tests with commonly encountered adulterants or excipients to quantitatively evaluate selectivity. Also, future work could employ explicit solvation models such as SMD or hybrid QM/MM simulations to validate adsorption stability and interaction dynamics in biological or aqueous media. Such extensions would complement the CPCM-based approach used here and further refine the predictive accuracy of the sensing mechanisms. These investigations will provide deeper insight into the robustness of the C_24_-based nanostructures under complex sample conditions and support the optimization of their real-world sensing performance.

## Conclusion

This study used advanced computational methods (e.g., NBO and NCI) to evaluate pure and doped C_24_ fullerenes (BC_23_ and ZnC_23_) as nanosensors for the detection of the narcotic drug Molly. In this regard, structural, electronic, optical, and adsorption properties were computationally studied, along with key sensing parameters such as recovery time, adsorption energy, electrical conductivity, and dipole moment.

Structurally, doping with boron and zinc not only changed the bond lengths and angles, but BC_23_ and ZnC_23_ showed higher cohesive energies (-144 and − 188 kcal/mol, respectively) than C_24_ (− 149 kcal/mol), indicating greater structural stability and a change in π electron mobility. In terms of electronic performance, BC_23_ and ZnC_23_ demonstrated smaller HOMO-LUMO energy gaps (5.68 eV and 5.24 eV) than C_24_ (6.12 eV), indicating increased chemical reactivity and better sensing potential. Upon complexation with Molly, BC23@Molly conformer 4 had the highest adsorption energy (− 18.19 kcal/mol), while ZnC_23_@Molly conformer 6 showed the fastest recovery time (3.8 × 10^− 4^ s) and highest electrical conductivity (2.78 × 10^9^ A m^− 2^), making it ideal for electrochemical sensing. Conversely, BC23@Molly presented stronger binding but slower desorption, indicating its suitability in filtration-based applications. Optically, BC_23_ showed the most pronounced colorimetric response with a significant redshift in λmax (from 444 to 660 nm) and a corresponding drop in excitation energy (1.87 eV), outperforming both C_24_ and ZnC_23_. NBO and second-order perturbation (E^2^) analysis further confirmed that BC_23_@Molly (conformer 4) exhibited the strongest π→π* and LP→π* transitions (21.13 and 55.97 kcal/mol, respectively), directly correlating with enhanced sensor sensitivity. Dipole moment analysis revealed that all complexes experienced substantial increases upon interaction with Molly (especially C_24_@Molly conformer 2 (15.68 D) and BC_23_@Molly conformer 4 (12.31 D)) which is beneficial for generating detectable electrical signals in polar environments. Finally, NCI and QTAIM analyses confirmed the presence of stabilizing and moderately strong van der Waals hydrogen bonds in the BC_23_@Molly and ZnC_23_@Molly complexes, indicating their robustness and selectivity.

According to the results, ZnC_23_ is recognized as the most efficient electrochemical sensor for Molly detection due to its fast response, high conductivity, and favorable binding energy. On the other hand, BC_23_ exhibits the most effective colorimetric sensing capabilities in the presence of Molly due to its significant optical shifts and strong donor-acceptor interactions.

## Data Availability

All data generated or analyzed during this study are included in this published article.
